# Supplementary material for: Message source effects on rejection and costly punishment of criticism across cultures
Source: Commun Psychol. 2025 Apr 16;3:64. doi: 10.1038/s44271-025-00248-z (PMC12003875; doi:10.1038/s44271-025-00248-z)
Supplement: Supplementary file 2 — Supplementary Information [file 44271_2025_248_MOESM2_ESM.pdf]

Supplementary Information 1: Comments

Note: The order of the comments and the order of the conditions was fully counterbalanced (randomized).

Comment 1

[source national flag]

The following comment was written by a former participant from [source nationality]

"In my experience, people from [target nationality] are unbelievable stubborn and inflexible. We (They) are really hard to talk to. Our (Their) own opinion counts more than anything, no matter how stupid or obviously wrong it is. I recommend to never even start a smart discussion with a person from [target nationality]. To win an argument, we (they) will stick with our (their) own stereotypes and wrong information."

Comment 2

[source national flag]

The following comment was written by a former participant from [source nationality]

"To be honest, people from [target nationality] are actually dim-witted. We (They) make believe that education and knowledge is important, but most of us (them) are rather unintelligent. And on top of that we (they) are most of the time easily tricked. It's clear that people from [target nationality] are rather simple minded, and it makes me sometimes so ashamed to see how dumb we (they) are."

Comment 3

[source national flag]

The following comment was written by a former participant from [source nationality]

"People from [target nationality] cannot be trusted. We (They) are just materialistic and arrogant and will do anything to fool you. We (They) really have no honour whatsoever and we (they) will not miss an opportunity to cheat. I know for a fact that people from [target nationality] also really like to cheat in sports and in education, that is why we (they) achieve such good results."

Comment 4

[source national flag]

The following comment was written by a former participant from [source nationality]

"People from [target nationality] are so rude and careless, and we (they) really only look out for ourselves (themselves). It is so embarrassing that we (they) are so obnoxious and standoffish, especially how we (they) think we (they) are superior, when most of us (them) are just really lazy. People from [target nationality] will find any means to avoid work and are happy to let others work for us (them)."

Supplementary Information 2: Additional Analyses

Robustness Checks Bonus Measure in the Chinese Sample

The bonus measure was displayed as “cent” instead of “lottery tickets” in Rounds 1 to 3 in the Chinese sample due to a programming error. Only including Round 4, the Message Source effect again emerged when China was criticized, $p = .043$, $d = .26$, *95% CI* [0.06; 0.50] but not when another country was criticized, $p = .817$, $d = 0.10$, *95% CI* [-0.08; 0.29]. Plotting bonus decisions (Figure SI1) moreover showed that responses to “cent” versus “lottery tickets” decisions did not differ and thus did not affect our results. Corroborating this view, the ISE has been observed on lottery ticket and cent measures in published registered reports.[19, 20]

Figure SI1. Boxplots with Violin-Plots: Message source (ingroup vs. outgroup) and Message target (ingroup vs. outgroup) by Round in the Chinese sample.

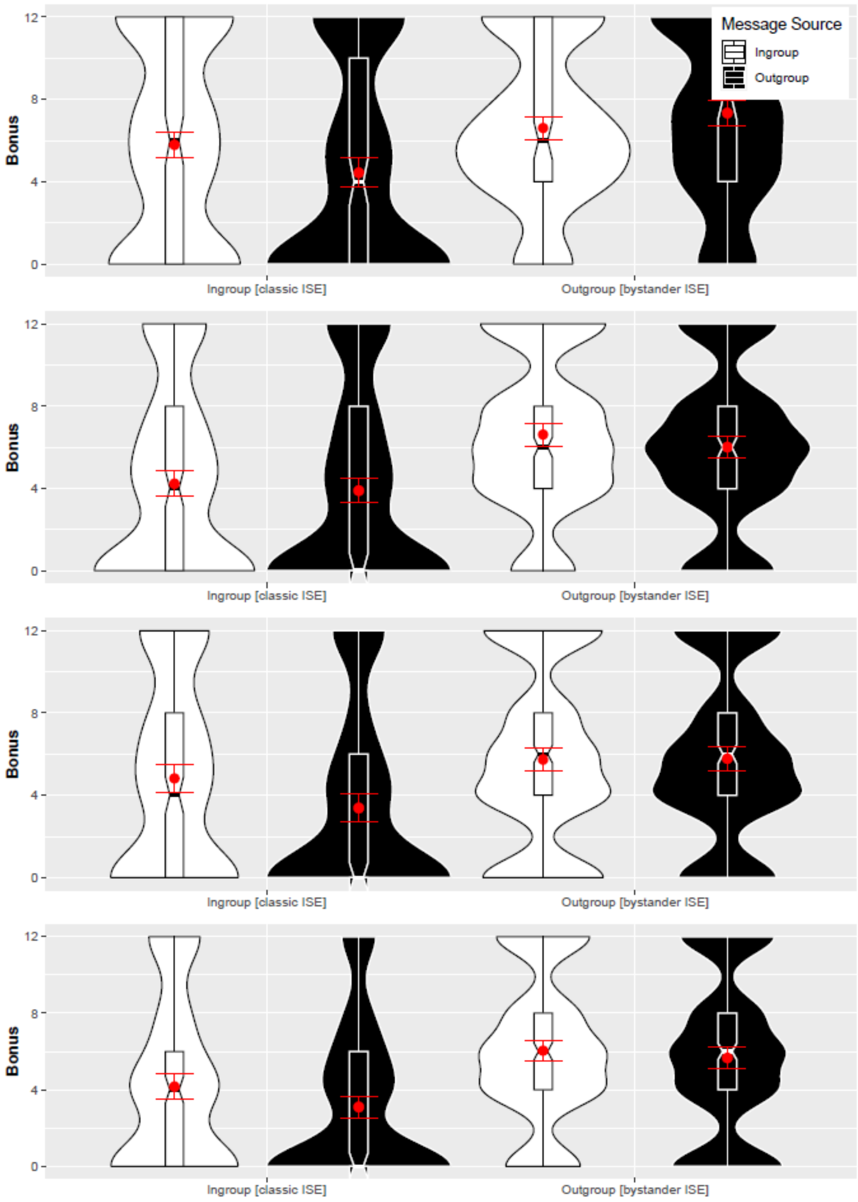

Note: Red dots with lines indicate means with 95% CIs. Bonus: Number of lottery tickets awarded to commenter. In Rounds 1-3 Chinese participants saw “ct” instead of “lottery tickets” when making their bonus decision; Round 4 was displayed correctly as “lottery tickets”.

Exploratory Analyses (E2): Appropriateness of Outgroup Criticism

Figure SI2. Johnson-Neyman-Plots for message source effect (ingroup vs. outgroup) conditional on reported appropriateness of criticizing outgroups (Norm2).

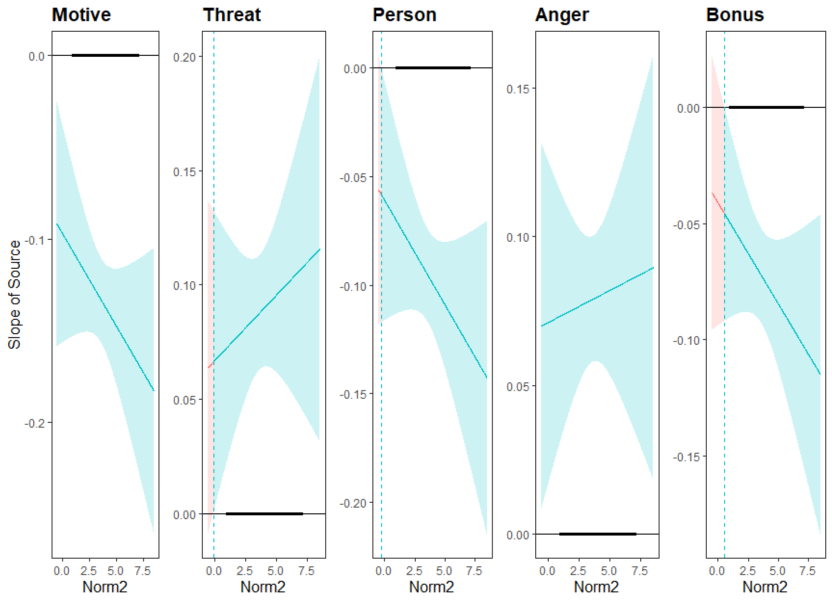

a)

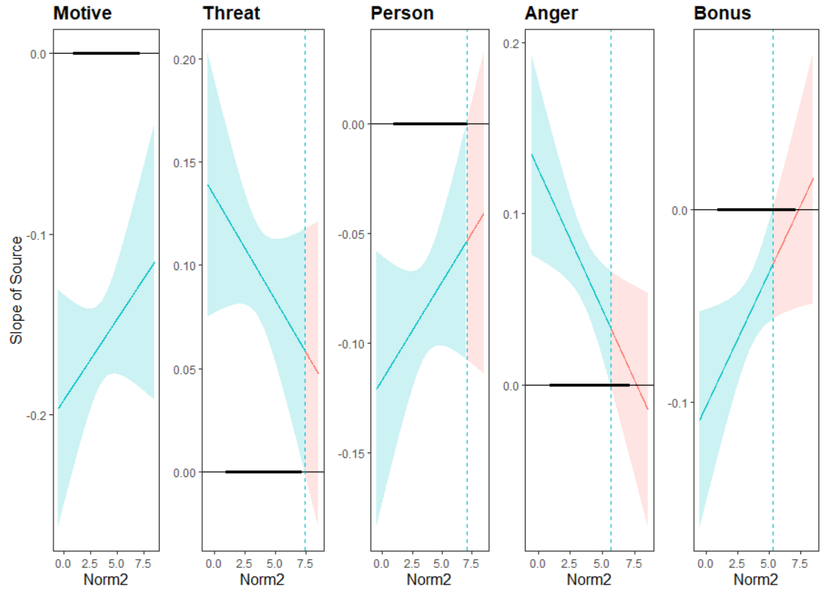

b)

Note: a) responses to criticism of participants' own national group (classic ISE); b) responses to criticism of other national group that participants were not a member of (bystander ISE). Message source effect is significant at α =.01 in green-shaded areas and non-significant in red-shaded areas. Bold horizontal line indicates observed values of norm endorsement. None of the respective Source × Norm2 interactions attained significance (Tables SI1 & SI2). Bonus: Number of lottery tickets awarded to commenter.

Table SI1. Regression results for message source effect (ingroup vs. outgroup) for a) criticism of participants' own national group (classic ISE) conditional on reported appropriateness of criticizing outgroups (Norm2).

Effect	*B*	*SE*	*t*	*df*	*p*
			Constructiveness		
(Intercept)	-0.568	0.047	-12.212	2205	<.001
Source	-0.097	0.023	-4.184	2205	<.001
Norm2	0.107	0.012	9.062	2205	<.001
Source:Norm2	-0.010	0.006	-1.713	2205	.087
			Threat		
(Intercept)	0.329	0.047	6.963	2205	<.001
Source	0.067	0.025	2.656	2205	.008
Norm2	-0.037	0.012	-3.050	2205	.002
Source:Norm2	0.006	0.006	0.897	2205	.370
			Person Evaluation		
(Intercept)	-0.571	0.047	-12.118	2205	<.001
Source	-0.061	0.021	-2.851	2205	.004
Norm2	0.105	0.012	8.777	2205	<.001
Source:Norm2	-0.010	0.005	-1.757	2205	.079
			Anger		
(Intercept)	0.420	0.048	8.712	2205	<.001
Source	0.071	0.021	3.344	2205	.001
Norm2	-0.034	0.012	-2.793	2205	.005
Source:Norm2	0.002	0.005	0.401	2205	.688
			Bonus Allocation		
(Intercept)	-0.368	0.051	-7.206	2205	<.001
Source	-0.041	0.020	-2.017	2205	.044
Norm2	0.065	0.013	4.968	2205	<.001
Source:Norm2	-0.009	0.005	-1.664	2205	.096

Table SI2. Regression results for message source effect (ingroup vs. outgroup) for criticism of other national group that participants were not a member of (bystander ISE) conditional on reported appropriateness of criticizing outgroups (Norm2).

Effect	*B*	*SE*	*t*	*df*	*p*
			Constructiveness		
(Intercept)	-0.228	0.049	-4.680	2205	<.001
Source	-0.192	0.023	-8.462	2205	<.001
Norm2	0.112	0.012	9.052	2205	<.001
Source:Norm2	0.009	0.006	1.553	2205	.120
			Threat		
(Intercept)	-0.044	0.049	-0.900	2205	.368
Source	0.134	0.022	6.053	2205	<.001
Norm2	-0.042	0.013	-3.348	2205	.001
Source:Norm2	-0.010	0.006	-1.805	2205	.071
			Person Evaluation		
(Intercept)	-0.272	0.050	-5.461	2205	<.001
Source	-0.116	0.022	-5.352	2205	<.001
Norm2	0.127	0.013	10.038	2205	<.001
Source:Norm2	0.009	0.006	1.604	2205	.109
			Anger		
(Intercept)	-0.175	0.048	-3.632	2205	<.001
Source	0.126	0.020	6.191	2205	<.001
Norm2	-0.033	0.012	-2.723	2205	.007
Source:Norm2	-0.017	0.005	-3.193	2205	.001
			Bonus Allocation		
(Intercept)	-0.113	0.050	-2.268	2205	.023
Source	-0.102	0.019	-5.257	2205	<.001
Norm2	0.068	0.013	5.359	2205	<.001
Source:Norm2	0.014	0.005	2.813	2205	.005

Exploratory Analyses (E3): Social Identification

Figure SI3. Johnson-Neyman-Plots for message source effect (ingroup vs. outgroup) for conditional on reported identification with participants' national group (Ident).

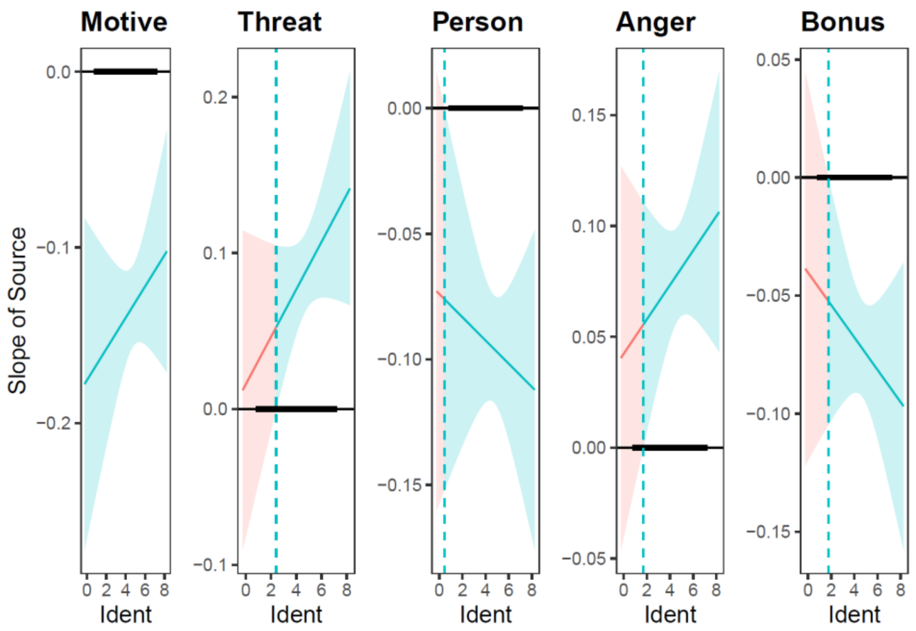

a)

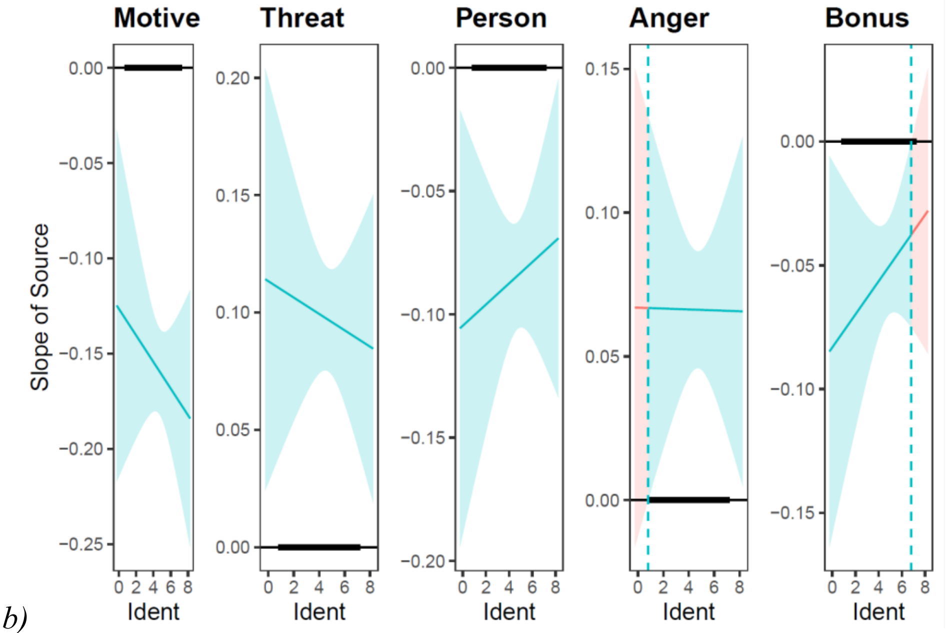

b)

Note: a) responses to criticism of participants' own national group (classic ISE); b) responses to criticism of other national group that participants were not a member of (bystander ISE). Message source effect is significant at α =.01 in green-shaded areas and non-significant in red-shaded areas. Bold horizontal line indicates observed values of identification. None of the respective Source × Identification interactions attained significance (Tables SI3 & SI4). Bonus: Number of lottery tickets awarded to commenter.

Table SI3. Regression results for message source effect (ingroup vs. outgroup) conditional on reported identification with participants' national group (Ident) in responsese to criticism of participants' own national group (classic ISE).

Effect	*B*	*SE*	*t*	*df*	*p*
Constructiveness					
(Intercept)	0.465	0.070	6.643	2205	<.001
Source	-0.176	0.035	-5.026	2205	<.001
Ident	-0.136	0.014	-9.521	2205	<.001
Source:Ident	0.009	0.007	1.243	2205	.214
Threat					
(Intercept)	-0.476	0.070	-6.805	2205	<.001
Source	0.016	0.038	0.417	2205	.677
Ident	0.142	0.014	9.946	2205	<.001
Source:Ident	0.015	0.008	1.962	2205	.050
Person Evaluation					
(Intercept)	0.513	0.070	7.275	2205	<.001
Source	-0.074	0.032	-2.291	2205	.022
Ident	-0.149	0.014	-10.304	2205	<.001
Source:Ident	-0.005	0.007	-0.699	2205	.485
Anger					
(Intercept)	-0.673	0.070	-9.679	2205	<.001
Source	0.042	0.032	1.322	2205	.186
Ident	0.205	0.014	14.412	2205	<.001
Source:Ident	0.008	0.007	1.183	2205	.237
Bonus Allocation					
(Intercept)	0.131	0.077	1.696	2205	.090
Source	-0.040	0.031	-1.307	2205	.191
Ident	-0.056	0.016	-3.550	2205	<.001
Source:Ident	-0.007	0.006	-1.086	2205	.278

Table SI4. Regression results for message source effect (ingroup vs. outgroup) conditional on reported identification with participants' national group (Ident) in responsese to criticism of other national group that participants were not a member of (bystander ISE).

Effect	*B*	*SE*	*t*	*df*	*p*
			Constructiveness		
(Intercept)	0.215	0.075	2.867	2205	.004
Source	-0.126	0.034	-3.699	2205	<.001
Ident	-0.008	0.015	-0.491	2205	.624
Source:Ident	-0.007	0.007	-0.997	2205	.319
			Threat		
(Intercept)	-0.320	0.074	-4.306	2205	<.001
Source	0.113	0.033	3.404	2205	.001
Ident	0.026	0.015	1.721	2205	.085
Source:Ident	-0.003	0.007	-0.511	2205	.609
			Person Evaluation		
(Intercept)	0.279	0.077	3.636	2205	<.001
Source	-0.105	0.033	-3.188	2205	.001
Ident	-0.019	0.016	-1.209	2205	.227
Source:Ident	0.004	0.007	0.640	2205	.522
			Anger		
(Intercept)	-0.514	0.072	-7.084	2205	<.001
Source	0.067	0.031	2.172	2205	.030
Ident	0.046	0.015	3.108	2205	.002
Source:Ident	-0.001	0.006	-0.025	2205	.980
			Bonus Allocation		
(Intercept)	0.063	0.076	0.831	2205	.406
Source	-0.083	0.029	-2.844	2205	.004
Ident	0.015	0.016	0.967	2205	.334
Source:Ident	0.007	0.006	1.118	2205	.264

Non Pre-Registered Exploratory Analyses: Culture

Table SI5. Regression results for self-reported cultural mindsets on difference score (ingroup vs. outgroup) for criticism of participants' own national group (classic ISE).

Effect	*B*	*SE*	*t*	*p*
		Constructiveness		
(Intercept)	-0.26	0.17	-1.56	.118
H	0.05	0.05	1.10	.273
HC	0.01	0.03	0.41	.683
HI	0.01	0.03	0.19	.851
VC	0.02	0.04	0.62	.534
VI	0.05	0.03	1.97	.050
		Threat		
(Intercept)	0.31	0.17	1.85	.065
H	-0.05	0.05	-1.05	.293
HC	0.02	0.04	0.47	.637
HI	-0.01	0.03	-0.20	.846
VC	0.00	0.04	-0.09	.932
VI	-0.09	0.03	-3.05	.002
		Person Evaluation		
(Intercept)	-0.44	0.15	-3.05	.002
H	0.04	0.04	0.97	.331
HC	0.00	0.03	-0.07	.946
HI	0.00	0.03	-0.18	.858
VC	0.03	0.03	1.06	.290
VI	0.09	0.02	3.88	<.001
		Anger		
(Intercept)	0.20	0.18	1.11	.266
H	-0.07	0.05	-1.49	.135
HC	0.06	0.04	1.69	.092
HI	0.03	0.03	1.03	.304
VC	-0.05	0.04	-1.31	.192
VI	-0.08	0.03	-2.76	.006
		Bonus Allocation		
(Intercept)	-1.09	0.37	-2.91	.004
H	0.15	0.10	1.44	.151
HC	0.05	0.08	0.63	.527
HI	0.07	0.06	1.04	.299
VC	-0.08	0.08	-0.96	.340
VI	0.17	0.06	2.80	.005

Note. Honor [H], Horizontal Collectivism [HC], Horizontal Individualism [HI], Vertical Collectivism [VC], Vertical Individualism [VI]

Table SI6. Regression results for self-reported cultural mindsets on difference score (ingroup vs. outgroup) for criticism of other national group that participants were not a member of (bystander ISE).

Effect	*B*	*SE*	*t*	*p*
		Constructiveness		
(Intercept)	0.82	0.16	5.00	<.001
H	-0.04	0.05	-0.88	.381
HC	0.01	0.03	0.16	.873
HI	0.00	0.03	0.12	.905
VC	0.02	0.04	0.44	.663
VI	-0.05	0.03	-1.83	.068
		Threat		
(Intercept)	-0.61	0.15	-4.08	<.001
H	-0.03	0.04	-0.74	.458
HC	-0.02	0.03	-0.78	.435
HI	0.00	0.03	0.18	.855
VC	0.05	0.03	1.52	.130
VI	0.07	0.02	2.71	.007
		Person Evaluation		
(Intercept)	0.67	0.15	4.52	<.001
H	0.02	0.04	0.42	.677
HC	0.02	0.03	0.70	.484
HI	-0.04	0.03	-1.70	.090
VC	-0.02	0.03	-0.74	.461
VI	-0.05	0.02	-2.22	.026
		Anger		
(Intercept)	-0.65	0.17	-3.76	<.001
H	0.02	0.05	0.36	.717
HC	-0.02	0.04	-0.58	.560
HI	-0.03	0.03	-1.03	.303
VC	0.06	0.04	1.49	.138
VI	0.06	0.03	2.25	.025
		Bonus Allocation		
(Intercept)	1.65	0.35	4.64	<.001
H	-0.06	0.10	-0.64	.526
HC	0.02	0.07	0.24	.813
HI	0.04	0.06	0.69	.489
VC	-0.03	0.08	-0.33	.743
VI	-0.24	0.06	-4.15	<.001

Note. Honor [H], Horizontal Collectivism [HC], Horizontal Individualism [HI], Vertical Collectivism [VC], Vertical Individualism [VI]

Table SI7. Regression results for message source effect (ingroup vs. outgroup) conditional on reported vertical individualism (VI) in response to criticism of participants' own national group (classic ISE).

Effect	*B*	*SE*	*t*	*df*	*p*
		Constructiveness			
(Intercept)	-0.63	0.06	-9.78	2205	<.001
Source	-0.01	0.03	-0.37	2205	.709
VI	0.10	0.01	7.29	2205	<.001
Source:VI	-0.03	0.01	-3.99	2205	<.001
		Threat			
(Intercept)	0.19	0.07	2.88	2205	.004
Source	-0.06	0.04	-1.79	2205	.073
VI	0.00	0.01	0.13	2205	.896
Source:VI	0.03	0.01	4.52	2205	<.001
		Person Evaluation			
(Intercept)	-0.48	0.07	-7.31	2205	<.001
Source	0.08	0.03	2.61	2205	.009
VI	0.07	0.01	4.61	2205	<.001
Source:VI	-0.04	0.01	-6.13	2205	<.001
		Anger			
(Intercept)	-0.40	0.07	-6.13	2205	<.001
Source	-0.04	0.03	-1.41	2205	.159
VI	0.16	0.01	11.15	2205	<.001
Source:VI	0.03	0.01	4.29	2205	<.001
		Bonus Allocation			
(Intercept)	-0.41	0.07	-5.79	2205	<.001
Source	0.05	0.03	1.93	2205	.053
VI	0.06	0.02	4.05	2205	<.001
Source:VI	-0.03	0.01	-4.71	2205	<.001

Table SI8. Regression results for message source effect (ingroup vs. outgroup) conditional on reported vertical individualism (VI) in response to for criticism of other national group that participants were not a member of (bystander ISE).

Effect	*B*	*SE*	*t*	*df*	*p*
		Constructiveness			
(Intercept)	-0.93	0.06	-14.57	2205	<.001
Source	-0.23	0.03	-7.51	2205	<.001
VI	0.25	0.01	18.10	2205	<.001
Source:VI	0.02	0.01	2.50	2205	.012
		Threat			
(Intercept)	0.36	0.07	5.44	2205	<.001
Source	0.20	0.03	6.52	2205	<.001
VI	-0.13	0.01	-8.73	2205	<.001
Source:VI	-0.02	0.01	-3.47	2205	.001
		Person Evaluation			
(Intercept)	-0.77	0.07	-11.56	2205	<.001
Source	-0.18	0.03	-6.05	2205	<.001
VI	0.22	0.01	14.99	2205	<.001
Source:VI	0.02	0.01	3.37	2205	.001
		Anger			
(Intercept)	-0.21	0.07	-3.18	2205	.002
Source	0.16	0.03	5.50	2205	<.001
VI	-0.02	0.01	-1.33	2205	.182
Source:VI	-0.02	0.01	-3.28	2205	.001
		Bonus Allocation			
(Intercept)	-0.59	0.07	-8.70	2205	<.001
Source	-0.19	0.03	-7.05	2205	<.001
VI	0.16	0.01	11.13	2205	<.001
Source:VI	0.03	0.01	5.32	2205	<.001

Supplementary Information 3: Gender Analyses

Table SI9. Mixed ANOVA results for each dependent variable across the sample including participant gender as an exploratory factor.

Dependent Measure	*F*	*df*	*p*	η^2
Message Source				
Constructiveness	509.55	(1, 2200)	<.001	.02
Message Threat	203.72	(1, 2200)	<.001	.01
Commenter Evaluation	220.58	(1, 2200)	<.001	.01
Anger	163.65	(1, 2200)	<.001	.06
Bonus Allocation	128.85	(1, 2200)	<.001	<.01
Message Target				
Constructiveness	369.08	(1, 2200)	<.001	.03
Message Threat	408.03	(1, 2200)	<.001	.04
Commenter Evaluation	388.17	(1, 2200)	<.001	.03
Anger	853.46	(1, 2200)	<.001	.09
Bonus Allocation	235.33	(1, 2200)	<.001	.02
Participant Location				
Constructiveness	140.25	(2, 2200)	<.001	.07
Message Threat	63.06	(2, 2200)	<.001	.03
Commenter Evaluation	70.98	(2, 2200)	<.001	.03
Anger	0.79	(2, 2200)	.453	<.01
Bonus Allocation	36.67	(2, 2200)	<.001	.02
Gender				
Constructiveness	12.06	(1, 2200)	<.001	<.01
Message Threat	4.62	(1, 2200)	.032	<.01
Commenter Evaluation	13.80	(1, 2200)	.002	<.01
Anger	1.81	(1, 2200)	.178	<.01
Bonus Allocation	5.93	(1, 2200)	.015	<.01
Message Source × Message Target				
Constructiveness	5.25	(1, 2200)	.022	<.01
Message Threat	0.75	(1, 2200)	.387	<.01
Commenter Evaluation	0.33	(1, 2200)	.563	<.01
Anger	0.50	(1, 2200)	.480	<.01
Bonus Allocation	1.67	(1, 2200)	.197	<.01
Message Source × Participant Location				
Constructiveness	2.85	(2, 2200)	.058	<.01
Message Threat	0.31	(2, 2200)	.731	<.01
Commenter Evaluation	1.07	(2, 2200)	.342	<.01
Anger	0.63	(2, 2200)	.535	<.01
Bonus Allocation	1.60	(2, 2200)	.201	<.01
Message Target × Participant Location				
Constructiveness	96.41	(2, 2200)	<.001	.02
Message Threat	56.87	(2, 2200)	<.001	.01
Commenter Evaluation	64.59	(2, 2200)	<.001	.01

Anger	103.76	(2, 2200)	<.001	.02
Bonus Allocation	40.10	(2, 2200)	<.001	.01
Message Source × Gender				
Constructiveness	3.65	(1, 2200)	.056	<.01
Message Threat	0.28	(1, 2200)	.597	<.01
Commenter Evaluation	3.68	(1, 2200)	.055	<.01
Anger	1.11	(1, 2200)	.292	<.01
Bonus Allocation	0.03	(1, 2200)	.861	<.01
Participant Location × Gender				
Constructiveness	2.02	(2, 2200)	.133	<.01
Message Threat	2.36	(2, 2200)	.095	<.01
Commenter Evaluation	0.68	(2, 2200)	.505	<.01
Anger	3.51	(2, 2200)	.030	<.01
Bonus Allocation	0.19	(2, 2200)	.825	<.01
Message Target × Gender				
Constructiveness	1.01	(1, 2200)	.316	<.01
Message Threat	0.80	(1, 2200)	.373	<.01
Commenter Evaluation	1.38	(1, 2200)	.240	<.01
Anger	1.81	(1, 2200)	.179	<.01
Bonus Allocation	0.23	(1, 2200)	.630	<.01
Message Source × Message Target × Participant Location				
Constructiveness	12.64	(2, 2200)	<.001	<.01
Message Threat	13.21	(2, 2200)	<.001	<.01
Commenter Evaluation	38.12	(2, 2200)	<.001	<.01
Anger	26.66	(2, 2200)	<.001	<.01
Bonus Allocation	20.74	(2, 2200)	<.001	<.01
Message Source × Message Target × Gender				
Constructiveness	0.06	(1, 2200)	.806	<.01
Message Threat	0.17	(1, 2200)	.677	<.01
Commenter Evaluation	0.04	(1, 2200)	.836	<.01
Anger	0.16	(1, 2200)	.686	<.01
Bonus Allocation	2.06	(1, 2200)	.151	<.01
Message Source × Gender × Participant Location				
Constructiveness	1.00	(2, 2200)	.368	<.01
Message Threat	0.33	(2, 2200)	.720	<.01
Commenter Evaluation	1.51	(2, 2200)	.220	<.01
Anger	0.80	(2, 2200)	.451	<.01
Bonus Allocation	1.93	(2, 2200)	.145	<.01
Gender × Message Target × Participant Location				
Constructiveness	0.41	(2, 2200)	.664	<.01
Message Threat	1.50	(2, 2200)	.223	<.01
Commenter Evaluation	0.98	(2, 2200)	.375	<.01
Anger	3.27	(2, 2200)	.038	<.01
Bonus Allocation	0.61	(2, 2200)	.545	<.01
Gender × Message Source × Message Target × Participant Location				
Constructiveness	0.29	(2, 2200)	.751	<.01

Message Threat	1.27	(2, 2200)	.280	<.01
Commenter Evaluation	0.09	(2, 2200)	.911	<.01
Anger	0.03	(2, 2200)	.969	<.01
Bonus Allocation	2.84	(2, 2200)	.059	<.01
