# Supplementary material for: Message source effects on rejection and costly punishment of criticism across cultures
Source: Commun Psychol. 2025 Apr 16;3:64. doi: 10.1038/s44271-025-00248-z (PMC12003875; doi:10.1038/s44271-025-00248-z)
Supplement: Supplementary file 3 — Reporting Summary [file 44271_2025_248_MOESM3_ESM.pdf]

nature portfolio

Corresponding author(s): J.L. Thürmer

Last updated by author(s): Mar 28, 2025

Reporting Summary

Nature Portfolio wishes to improve the reproducibility of the work that we publish. This form provides structure for consistency and transparency in reporting. For further information on Nature Portfolio policies, see our Editorial Policies and the Editorial Policy Checklist.

Statistics

For all statistical analyses, confirm that the following items are present in the figure legend, table legend, main text, or Methods section.

n/a	Confirmed	
☐	☒	The exact sample size (*n*) for each experimental group/condition, given as a discrete number and unit of measurement
☐	☒	A statement on whether measurements were taken from distinct samples or whether the same sample was measured repeatedly
☐	☒	The statistical test(s) used AND whether they are one- or two-sided<br>*Only common tests should be described solely by name; describe more complex techniques in the Methods section.*
☒	☐	A description of all covariates tested
☐	☒	A description of any assumptions or corrections, such as tests of normality and adjustment for multiple comparisons
☐	☒	A full description of the statistical parameters including central tendency (e.g. means) or other basic estimates (e.g. regression coefficient) AND variation (e.g. standard deviation) or associated estimates of uncertainty (e.g. confidence intervals)
☐	☒	For null hypothesis testing, the test statistic (e.g. *F*, *t*, *r*) with confidence intervals, effect sizes, degrees of freedom and *P* value noted<br>*Give P values as exact values whenever suitable.*
☒	☐	For Bayesian analysis, information on the choice of priors and Markov chain Monte Carlo settings
☒	☐	For hierarchical and complex designs, identification of the appropriate level for tests and full reporting of outcomes
☐	☒	Estimates of effect sizes (e.g. Cohen's *d*, Pearson's *r*), indicating how they were calculated

Our web collection on statistics for biologists contains articles on many of the points above.

Software and code

Policy information about availability of computer code

Data collection	formR was used and is credited in the paper
Data analysis	R was used and is credited in the paper

For manuscripts utilizing custom algorithms or software that are central to the research but not yet described in published literature, software must be made available to editors and reviewers. We strongly encourage code deposition in a community repository (e.g. GitHub). See the Nature Portfolio guidelines for submitting code & software for further information.

Data

Policy information about availability of data

All manuscripts must include a data availability statement. This statement should provide the following information, where applicable:

- Accession codes, unique identifiers, or web links for publicly available datasets
- A description of any restrictions on data availability
- For clinical datasets or third party data, please ensure that the statement adheres to our policy

All data needed to evaluate the conclusions in the paper are present in the paper and/or the Supplementary Materials. Our pre-registration is available at https://www.psycharchives.org/en/item/74926c0c-425b-4210-816c-2b8232566fa0. Raw data and analyses are available at https://osf.io/djkz8/. Data collection of the present study was funded by PsychLab, a service of the Leibniz Institute for Psychology (ZPID).

Human research participants

Policy information about studies involving human research participants and Sex and Gender in Research.

Reporting on sex and gender	leaving N = 2207 for analysis (1245 men, 961 women, 1 other/non-binary; Mage = 46.87, SD = 11.98, range [17; 75]; Canada n = 666, China n = 786, Japan n = 755)<br>Exploratory gender analyses yielded no consistent effects and are reported.
Population characteristics	s.o.
Recruitment	We collected data between 03/17/2023 and 03/29/2023 using the professional panel provider Respondi (respondi.com) who was hired directly by ZPID's service PsychLab ONLINE.
Ethics oversight	The University of Salzburg internal review board approved this study (GZ 10/2020).

Note that full information on the approval of the study protocol must also be provided in the manuscript.

Field-specific reporting

Please select the one below that is the best fit for your research. If you are not sure, read the appropriate sections before making your selection.

☐ Life sciences ☒ Behavioural & social sciences ☐ Ecological, evolutionary & environmental sciences

For a reference copy of the document with all sections, see nature.com/documents/nr-reporting-summary-flat.pdf

Behavioural & social sciences study design

All studies must disclose on these points even when the disclosure is negative.

Study description	We used a 2 Comment Target (participants' own group vs. other group) × 2 Comment Source (criticized group vs. other group) within-participants design to manipulate comment source and comment target. Within designs attain a high power and are appropriate in the current context,51 as demonstrated in previous ISE research.20
Research sample	We collected data between 03/17/2023 and 03/29/2023 using the professional panel provider Respondi (respondi.com) who was hired directly by ZPID's service PsychLab ONLINE.
Sampling strategy	We collected data between 03/17/2023 and 03/29/2023 using the professional panel provider Respondi (respondi.com) who was hired directly by ZPID's service PsychLab ONLINE.
Data collection	Participants responded to the panel provider's study advertisements by clicking on a link to a web-based survey in formR.73 Participants were told that we needed their help in rating comments by previous participants in a study on people's thoughts about international teamwork in times of globalization and to learn about their personal opinion. Participants read critical comments describing people from the respective target country as difficult to work with.
Timing	We collected data between 03/17/2023 and 03/29/2023
Data exclusions	As preregistered, we excluded 194 participants for giving the same response on at least one questionnaire page ("straight liners") and 61 for participating repeatedly (as indicated by their IP address), leaving N = 2207 for analysis (1245 men, 961 women, 1 other/non-binary; Mage = 46.87, SD = 11.98, range [17; 75]; Canada n = 666, China n = 786, Japan n = 755).
Non-participation	s.o.
Randomization	The order of the comments and order of the conditions was fully counterbalanced (randomized) by the survey software formR73 and did not affect the reported results.

Reporting for specific materials, systems and methods

We require information from authors about some types of materials, experimental systems and methods used in many studies. Here, indicate whether each material, system or method listed is relevant to your study. If you are not sure if a list item applies to your research, read the appropriate section before selecting a response.

Materials & experimental systems

n/a	Involved in the study
☒	☐ Antibodies
☒	☐ Eukaryotic cell lines
☒	☐ Palaeontology and archaeology
☒	☐ Animals and other organisms
☒	☐ Clinical data
☒	☐ Dual use research of concern

Methods

n/a	Involved in the study
☒	☐ ChIP-seq
☒	☐ Flow cytometry
☒	☐ MRI-based neuroimaging
